# High frequency of *Taenia solium* antigen positivity in patients admitted for neurological disorders in the Rural Hospital of Mosango, Democratic Republic of Congo

**DOI:** 10.1186/s12879-021-06032-8

**Published:** 2021-04-17

**Authors:** Deby Mukendi, Jean-Roger Lilo Kalo, Pascal Lutumba, Barbara Barbé, Jan Jacobs, Cedric P. Yansouni, Sarah Gabriël, Pierre Dorny, François Chappuis, Marleen Boelaert, Andrea S. Winkler, Kristien Verdonck, Emmanuel Bottieau

**Affiliations:** 1 Institut National de Recherche Biomédicale, Kinshasa, Democratic Republic of the Congo; 2 Départment de Neurologie, Université de Kinshasa, Kinshasa, Democratic Republic of the Congo; 3 Department of Clinical Sciences, Institute of Tropical Medicine, Antwerp, Belgium; 4 Department of Microbiology and Immunology, KU Leuven, Leuven, Belgium; 5 JD MacLean Centre for Tropical Diseases, McGill University Health Centre, Montreal, Canada; 6 Department of Veterinary Public Health, Faculty of Veterinary Medicine, Ghent University, Ghent, Belgium; 7 Department of Biomedical Sciences, Institute of Tropical Medicine, Antwerp, Belgium; 8 Division of Tropical and Humanitarian Medicine, Geneva University Hospitals and University of Geneva, Geneva, Switzerland; 9 Department of Public Health, Institute of Tropical Medicine, Antwerp, Belgium; 10 Center for Global Health, Department of Neurology, Technical University of Munich, Munich, Germany; 11 Center for Global Health, University of Oslo, Oslo, Norway

**Keywords:** Neurocysticercosis, *Taenia solium*, Neurology, Serological test, Democratic Republic of Congo, Cross-sectional study

## Abstract

**Background:**

The epidemiology of human cysticercosis and neurocysticercosis, caused by the larval stage of the pork tapeworm *Taenia solium*, is not well known in the Democratic Republic of Congo (DRC). Within a multicenter etiological and diagnostic study conducted by the NIDIAG consortium (“Better Diagnosis for Neglected Infections”) and investigating several challenging syndromes, we consecutively evaluated from 2012 to 2015 all patients older than 5 years presenting with neurological disorders (neurology cohort) and with fever > 7 days (persistent fever cohort) at the rural hospital of Mosango, province of Kwilu, DRC. In both cohorts, etiological diagnosis relied on a systematic set of reference laboratory assays and on pre-established clinical case definitions. No neuroimaging was available in the study hospital. In this study, we determined the frequency of *T. solium* infection in both cohorts and explored in the neurology cohort its association with specific neurological presentations and final etiological diagnoses.

**Methods:**

We conducted a post-hoc descriptive and analytic study on cysticercosis in the neurology and persistent fever cohorts, based on the presence in serum samples of circulating *T. solium* antigen using the B158/B60 enzyme-linked immunosorbent assay (ELISA) and of cysticercosis IgG using the LDBIO Cysticercosis Western Blot IgG assay.

**Results:**

For the neurology cohort, 340 samples (of 351 enrolled patients) were available for analysis (males: 46.8%; mean age: 38.9 years). *T. solium* antigen positivity was found in 43 participants (12.6%; 95% confidence interval [CI] 9.3–16.7%), including 9 of 60 (15%) patients with epilepsy. Among the 148 samples available from the persistent fever cohort (males: 39.9%; mean age: 19.9 years), 7 were positive in the *T. solium* antigen ELISA (4.7%; 95% CI 1.9–9.5%; *P* = 0.009 when compared to the neurology cohort). No significant association was found within the neurology cohort between positivity and clinical presentation or final diagnoses. Of note, the IgG antibody-detecting assay was found positive in only four (1.3%) of the participants of the neurology cohort and in none of the persistent fever cohort.

**Conclusions:**

*T. solium* antigen positivity was found in at least 10% of patients admitted with neurological disorders in the Kwilu province, DRC, with no specific pattern of presentation. Further neuroimaging studies should be used to confirm whether neurocysticercosis is prevalent in this region.

## Background

Cysticercosis is an infection caused by the metacestode larval stage of the tapeworm *Taenia solium* that affects pigs and humans, in areas where sanitation and veterinary control are poor. Pigs act as intermediate hosts by ingesting *T. solium* eggs released in human feces. Humans are the only definitive hosts able to harbor adult tapeworms in the intestines, that may cause a rather mild illness called taeniasis. Humans, however, may also develop cysticercosis after ingestion of eggs, if the food/water or environment is contaminated with human fecal material, or by self-infection in case of adult worm carriage [1]. Larval cysts (also called cysticerci) may settle in different tissues (muscles, skin) but are preferentially localized in the central nervous system (brain and spinal cord), causing neurocysticercosis, a disease associated with major morbidity, poor quality of life and significant cost for the health-care system, particularly in low-resource settings [2, 3]. Depending on the number, size, stage, and location of the cysticerci as well as the host’s immune response, neurocysticercosis may cause a wide variety of neurological symptoms and signs, including seizures, headaches, focal deficits, psychiatric manifestations and cognitive impairment [4–6]. On the other hand, some of the people carrying cysticerci in the central nervous system or in other locations may remain fully asymptomatic [2]. An accurate diagnosis of neurocysticercosis almost always requires a combination of epidemiological, clinical, imaging and serological information, as very few elements are fully diagnostic in isolation. Definitions of probable or definitive diagnosis of neurocysticercosis are based on a set of neuroimaging and clinical/exposure major and minor criteria, which have been recently revised by Del Brutto et al. [7]. Major neuroimaging criteria include cystic lesions, enhancing lesions, multilobulated cysts (all these lesions are usually referred to as active neurocysticercosis) and calcifications (referred to as inactive neurocysticercosis, in the absence of other active lesions). Well-standardized immunoassays are used as major “clinical/exposure criteria” and may detect either circulating *T. solium* antigens, reflecting the presence of living cysticerci in any tissue (current/active cysticercosis), or antibodies, which may be present in case of exposure and in current or past infection [8]. For this reason, sensitivity of antigen-based assays is usually lower than that of antibody-based assays for diagnosing cysticercosis [9]. Of note, in low-resource settings, brain imaging and most immunoassays are usually not available or not affordable, making the definitive diagnosis of neurocysticercosis rarely possible [3, 10] .

Neurological disorders account for approximately 10% of all admissions in African rural hospitals [11–13]. However, the etiological spectrum has been hardly studied so far because of lack of diagnostic facilities [14]. To fill this knowledge gap, we investigated the infectious etiologies of neurological disorders in the rural hospital of Mosango, province of Kwilu, Democratic Republic of Congo (DRC) [15], as part of the NIDIAG project(“Better diagnosis of Neglected Infectious Diseases”, https://nidiag.eu/) that investigated the etiologies of several challenging syndromes in various tropical countries. In this rural DRC setting where neuroimaging is not available, the contribution of neurocysticercosis in the neurological case load is unknown. *T. solium* is highly endemic in the Bas Congo province, west of the capital Kinshasa, and is suspected to be present in other parts of the country as well [15]. The aim of this study was to determine the frequency of positivity of antigen- and antibody-based *T. solium* immunoassays in the clinical samples of the participants of the NIDIAG neurology study (neurology cohort), as well as to explore the association with the clinical presentation and final established diagnosis. A secondary objective was to determine the frequency of immunoassay positivity in another NIDIAG cohort of patients evaluated for persistent fever (> 7 days) in the same study hospital and during the same period (persistent fever cohort). This cohort of patients with non-neurological symptoms as used as comparator.

## Methods

### Study participants and setting

We conducted a post-hoc descriptive and analytic study for cysticercosis in the serum samples of the participants of the neurology and persistent fever cohorts, based on the presence of *T. solium* antigen and antibodies. The rationale, objective, design, study population and setting of the NIDIAG neurology study have been described in detail previously [15]. Briefly, all consecutive children older than 5 years and adults presenting with neurological disorders to the “Hôpital Général de Référence” (HGR) of Mosango, province of Kwilu were prospectively enrolled and evaluated. Entry criteria included any of the following: altered state of consciousness; change in sleep pattern; cognitive decline; changes in personality/behavior; recent (< 2 weeks) epileptic seizure; recent, severe and progressive headache; meningism; new onset cranial nerve lesion; new onset sensory-motor focal deficit; and new onset gait/walking disorders. Participants underwent a systematic diagnostic workup including a microscopic examination of cerebrospinal fluid (CSF) unless this was contra-indicated. Neuro-imaging diagnostic methods were not available at the study site. A pre-established set of severe and treatable infections of the central nervous system (CNS) were considered as “targeted infections” for the NIDIAG study. They comprised human African trypanosomiasis, malaria, bacterial meningitis, unspecified meningo-encephalitis, HIV-related neurological disorders, CNS tuberculosis and neurosyphilis. These targeted infections were searched for systematically with reference methods (either on site, or in reference laboratories in Kinshasa, DRC or Antwerp, Belgium). Other diagnoses (of non-targeted conditions) were made in 2016 by a panel of experts in neurology and infectious diseases, who reviewed the complete clinical case files and all laboratory results and followed pre-established case definitions [15]. Three hundred fifty-one patients were enrolled in the NIDIAG neurology study between 2012 and 2015.

In parallel, from 2013 to 2015, 300 children > 5 years and adults presenting to the same study hospital with persistent fever (defined as fever reported or documented for more than 7 days) were prospectively included in the NIDIAG persistent fever study. They also underwent a systematic diagnostic work-up to explore a pre-established specific set of targeted severe and treatable etiologies.

### Samples and laboratory procedures

All sera of the NIDIAG studies were shipped on dry ice to the Institute of Tropical Medicine, Antwerp and stored in the biobank at − 80 °C. For the present study, we selected all available serum samples from the entire NIDIAG neurology cohort and a subset of 150 samples from the persistent fever cohort (every other consecutive participant). In April 2018, these serum samples were processed for the detection of circulating cysticercal antigens and antibodies. The presence of circulating cysticercal antigen was measured in the serum using the in house B158/B60 enzyme-linked immunosorbent assay (Ag-ELISA). The optical density (OD) for each sample was divided by a cut-off OD value to obtain a ratio. The sample was considered positive for cysticercal antigens if the ratio was greater than 1.0 [16, 17]. At this cut-off, the assay has a sensitivity of 90 to 100% to detect current cysticercosis and a specificity of 83 to 98% [10, 18]. To determine the presence of antibodies against *T. solium* infection, a commercial Western Blot assay using lentil-lectin purified glycoprotein antigens was used (Cysticercosis WB IgG, LDBIO Diagnostics, Lyon, France). This assay is considered to provide information on exposure to/current and past infection with *T. solium*, with a sensitivity of 98% and a specificity of 100% according to the manufacturer [19]. A result is considered positive if a minimum of two of the five bands react with the serum sample in the Western Blot. The laboratory technician who performed both *T. solium* assays was blinded to all clinical and diagnostic information. When the available serum volume was small, the antigen-detecting assay was prioritized.

### Data analysis and reporting

Data were analyzed with SPSS software, version 25.0 (SPSS, Chicago, IL, USA). For both the NIDIAG neurology and persistent fever cohorts, the frequency of *T. solium* positivity was determined with a 95% confidence interval (CI) and stratified by age group. Within the NIDIAG neurology cohort, the association of *T. solium* antigen positivity with clinical features and final diagnosis was also assessed. Baseline epidemiological characteristics and frequency data were compared between cohorts. Chi-square test or Fisher’s exact test were used for categorical variables. Continuous variables were compared using parametric tests. All tests were two-sided and *p* values < 0.05 were considered statistically significant.

### Ethical aspects

The study protocol was approved by the Institutional Review Board of the Institute of Tropical Medicine, Antwerp, Belgium and by the Ethics Committees of the University of Antwerp, Belgium (reference 11/50/400; 2012), and the Public Health School of Kinshasa, DRC (reference ESP/CE/016/2012). The study was registered at clinicaltrials.gov (identifier NCT01589289). Before enrolment, participants or their legal guardians gave written informed consent for study participation and for the future use of their stored clinical samples for further etiological or diagnostic research. For children between 12 and 18 years, in addition to the parental consent, informed assent was necessary for inclusion, in line with the DRC law.

## Results

Serum samples were available for 340 out of the 351 participants (97%) of the NIDIAG neurology cohort (Table 1). The mean age of this study population was 38.9 years [standard deviation: 17.7; range: 6–78); and the male-to-female ratio was 0.88 (159/181). Duration of neurological symptoms was more than 2 weeks in 190 (55.9%) of the evaluated patients and almost half had prior contact with a primary health care facility. Severe headache was the most frequent complaint, followed by gait/walking disorders and seizures. Confirmed targeted infections accounted for 84 (24.7%) of the final diagnoses while 156 (45.9%) cases were classified as non-communicable conditions, including mainly epilepsy and psychiatric disorders (Table 1). Death occurred in 26 patients (7.6%). The antigen-detecting assay was positive in 43/340 (12.6%; 95% CI 9.3–16.7%) participants of the NIDIAG neurology cohort. The antibody-detecting assay could be done in 314 samples, and was positive in four of them (1.3%; 95% CI 0.5–3.2%). All four antibody-positive cases were also *T. solium* antigen positive.

**Table 1:** Baseline features, final diagnoses and outcome of 340 patients of the NIDIAG neurology cohort with available *T. solium* antigen results

	Total participants evaluated (*n* = 340)	*TS* antigen negative participants (*n* = 297)	*TS* antigen positive participants (*n* = 43)	*P*
*Features at presentation*	n (%)	n (%)	n (%)	
Male sex	159 (46.8)	137 (46.1)	22 (51.2)	0.5
Female sex	181 (53.2)	160 (53.8)	21 (48.8)	0.5
Age > 20 years	270 (79.4)	231 (77.8)	39 (90.7)	0.05
Prior contact with primary care facility	159 (46.8)	141 (47.5)	18 (41.9)	0.4
Neurological symptoms > two weeks	190 (55.9)	168 (56.6)	22 (51.2)	0.5
Fever reported/documented	99 (29.1)	89 (30.0)	10 (23.3)	0.3
Severe headache	156 (45.9)	136 (45.8)	20 (46.5)	0.9
Severe headache without fever	101 (29.7)	86 (29.0)	15 (34.9)	0.4
Gait/walking disorders	97 (28.5)	86 (29.0)	11 (25.6)	0.6
Seizure	84 (24.7)	73 (24.6)	11 (25.6)	0.8
Focal sensory-motor deficit	77 (22.6)	67 (22.6)	10 (23.3)	0.9
Cognitive and/or behavior disturbance	72 (21.2)	60 (20.2)	12 (27.9)	0.2
Altered state of consciousness	69 (20.3)	61 (20.5)	8 (18.6)	0.7
*Final diagnoses*	n (%)	n (%)	n (%)	
Confirmed targeted infections	84 (24.7)	76 (25.6)	8 (18.6)	0.3
Confirmed and suspected infections	119 (35.0)	107 (36.0)	12 (27.9)	0.2
Non-communicable conditions	156 (45.9)	134 (45.1)	22 (51.2)	0.4
Epilepsy	60 (17.6)	51 (17.2)	9 (20.9)	0.5
Psychiatric disorders	53 (15.6)	47 (15.8)	6 (14.0)	0.7
Myelo-radiculo-neuropathic syndromes	37 (10.9)	30 (10.1)	7 (16.3)	0.2
Cerebrovascular accident	23 (6.8)	20 (6.7)	3 (7.0)	0.9
*Outcome*	n (%)	n (%)	n (%)	
Death	26 (7.6)	23 (7.7)	3 (7.0)	0.8

*TS* denotes *Taenia solium*

Within the neurology cohort, there were no differences regarding presenting features and final grouped or single diagnoses between *T. solium* antigen-positive and -negative participants (Table 1). There was only a trend for higher frequency of patients older than 20 years in the *T. solium* antigen-positive group (*P* = 0.05).

In the neurology cohort, the frequency of *T. solium* antigen positivity was 12.6% in patients with reported/documented fever and 13.7% in patients without fever (*P* = 0.2). Slight non-significant variations in frequency were observed between the presenting neurological symptoms in the whole cohort or when restricted to non-febrile patients. *T. solium* antigen was positive in 8/84 (9.5%) of patients with confirmed targeted infection and in 22/156 (14%) of those finally diagnosed with non-communicable conditions, but this difference was not statistically significant (*P* = 0.3). Antigen positivity was observed in 9 of 60 (15%) patients diagnosed with epilepsy.

Of the 150 samples obtained from the patients with persistent fever, 148 could be analyzed with both antigen- and antibody-based tests. The male-to-female ratio was lower than in the neurology cohort (0.66; 59/89), but this difference was not statistically significant (Table 2). In contrast, mean age was 19.9 years (standard deviation: 16.2; range: 6–72) in this cohort, and was significantly lower than that of the neurology cohort (*p* < 0.001). *T. solium* antigen was positive in 7/148 (4.7%; 95% CI 1.9–9.5%). No single case of positive *T. solium* antibody assay was found. As shown in Table 2, the frequency of *T. solium* antigen positivity was significantly higher in the neurology compared to the persistent fever cohort (*P* = 0.009; odds ratio 2.9; 95% confidence interval 1.3–6.6). When we stratified by age, the frequency of positivity remained higher in the neurology cohort, but was only statistically significant for subjects over 40 years of age.

**Table 2:** Epidemiological features and frequency of *T. solium* antigen and antibody positivity in the neurology and persistent fever cohorts evaluated at the rural hospital of Mosango, Democratic Republic of Congo

	Neurological cohort (*n* = 340)	Persistent fever cohort (*n* = 148)	*P*
Epidemiological data			
Male sex, n (%)	159 (47)	59 (40)	0.1
Female sex, n (%)	181 (53)	89 (60)	0.1
Mean age (SD), years	39.9 (17.7)	19.9 (16.2)	< 0.001
Positive *T. solium* antigen assay			
Total group, n (%)	43 (12.6)	7 (4.7)	0.009
Age group ≤20 years, n (%)	4 (1.1)	3 (2)	0.4
Age group 21–40 years, n (%°	17 (5)	3 (2)	0.1
Age group > 40 years, n (%)	22 (6.4)	1 (0.7)	0.006
Positive *T. solium* antibody assay			
Total group n/n (%)	4/314 (1.3)	0/148 (0)	0.3

*Note*: *SD* denotes standard deviation

## Discussion

In this post-hoc analysis of a prospective cohort of patients presenting with neurological disorders in a rural hospital of Central Africa, we found that about 13% had evidence of circulating *T. solium* antigen in serum. There were no clear associations between presenting symptoms or final diagnoses and positivity *for T. solium* antigen. The frequency of antigen positivity was significantly higher in the neurology cohort than in the “comparator” persistent fever cohort (approximatively 5%), but a statistical difference was only observed in the subgroup of participants older than 40 years. Surprisingly, the antibody-based immunoassay was positive in only 1.3% of the neurological patients and in none of the persistent fever cohort.

This exploratory study has many limitations, most of which were largely acknowledged in previous publications reporting on the NIDIAG findings in DRC [15, 20, 21]. The source NIDIAG study was not initially designed to investigate risk factors for neurocysticercosis since the presence of this disease had not been reported in humans in the region so far. The use of patients rather than a sample of the general population is another limitation of this post-hoc analysis as it does not enable the full magnitude of the underlying problem to be estimated. A complete diagnostic workup was restricted to a set of targeted infections for both the neurology and the persistent fever studies. In particular for the neurology study, the etiological workup was limited by the absence of advanced neurological investigations in this low-resource hospital. Consequently, no causative link can be formally established between the serological markers of *T. solium* infection and the neurological presentation in the absence of brain/spinal imaging that could demonstrate cysticerci. Also, the persistent fever cohort was opportunistically used for comparison, but cannot be considered as a “control” group as such since the distribution of age groups was different. Finally, we acknowledge that the surprisingly low prevalence of *T. solium* antibodies in the neurology cohort may be considered an important weakness. Indeed, one would have expected a proportion of antibody-positive participants, indicating exposure to *T. solium* eggs, larger than that of antigen positivity. A possible explanation is that some degradation of the *T. solium* immunoglobulins has inflated the false negative rate when the antibody assay was performed in 2018, while blood sampling took place between 2012 and 2015. However, transport and storage were rigorously monitored during the study and other serological investigations on the same stored samples did provide plausible results [22]. Alternatively, this may have been related to differences in the circulating strains of *T solium*, parasitic load or host characteristics compared to settings where the antibody-detection assay was validated [23]. A failure of the LDBIO assay itself is unlikely since it performed well with positive and negative controls. However, sensitivity of this commercial assay has been reported in one study as substantially inferior to that of the reference Centers of Disease Control-developed enzyme-linked electroimmunotransfer blot (CDC-EITB), i.e. 13.3% versus 52.2% among 23 CT-confirmed neurocysticercosis cases tested [24]. On the other hand, in a recent Mexican study on 58 NCC patients, the sensitivity of the LDBIO EITB was slightly higher (71.4%) than that of the CDC-EITB (66.1%) [25]. These discrepancies between reference EITB test sensitivities need further investigation on a larger number of samples. In general, other types of commercial antibody-based-assays such as ELISA have limited sensitivities and specificities for diagnosing neurocysticercosis [26].

The African continent reports the highest burden of human cysticercosis in the world, with pooled prevalence of circulating *T. solium* antigens estimated at 7.30% (95% CI 4.23–12.31) and of antibodies at 17.37% (95%CI 3.33–56.20) in community-based epidemiological surveys [25]. In DRC, the epidemiology of human and porcine cysticercosis is poorly understood. Some surveys in the animal markets of Kinshasa have revealed active cysticercosis in pigs originating from different DRC provinces [18], including in more than 30% of those coming from Bandundu (the former name of the Kwilu province). Very high prevalence of human cysticercosis (proportion of *T. solium* antigen positivity up to 21%) has been reported in the only community-based survey performed so far, in the province of Bas Congo [16].

It is generally accepted that a higher proportion of neurocysticercosis cases is usually found in the older age categories [27, 28]. We found some similar trend when looking at *T. solium* antigen positivity, but cannot strongly support this observation since age categories were not evenly distributed in both cohorts and we did not specifically investigate neurocysticercosis. In contrast, we found no evidence of a difference in gender prevalence in our study. This agrees with findings reported in Bolivia [29], but contrasts with data from Guatemala and Tanzania where a higher prevalence was found in females [30, 31], and with findings in DRC and Vietnam, which showed a higher prevalence in males [32, 33]. These differences might be explained by different living conditions and the distribution of household tasks between genders in those countries.

The prevalence of neurocysticercosis remains unknown in most low-resource settings, since neuroimaging is required to establish the diagnosis [34]. The frequency of probable and definitive neurocysticercosis has been mainly investigated in patients with epilepsy, in whom the pooled estimate obtained from brain CT Scan-based studies was 30%, globally [34] and in Latin America [29]. Additional studies performed later on in Africa revealed that the proportion of neurocysticercosis in patients with epilepsy varied a lot according to the setting and could range from 15 to 50% [24, 31, 32].

Diagnostic accuracy studies of antigen- and antibody-based assays for the specific diagnosis of neurocysticercosis (based on CT findings) in Africa have been limited to small case series. Using Del Brutto’s definition of probable or definitive neurocysticercosis in patients evaluated for epilepsy in Zambia, Gabriël et al. found a sensitivity of 44% (15 positive results/34 cases) for *T. solium* antigen, when using a ratio of 1.0 as the cutoff, and a specificity of 90% (10). When restricting the analysis to patients with active neurocysticercosis (i.e. presence of viable cystic lesions, *n* = 6), the sensitivity was 100% and the specificity 84%. Antibody-based Western Blot assays are usually reported as highly sensitive for neurocysticercosis in patients with more than one viable cystic lesion, but performance is much lower in cases of single lesions or of calcifications only. The specificity of antibody-based tests for neurocysticercosis is variable since distinction with previous exposure or cured infection cannot be made [23]. As already mentioned, diagnostic performance of antibody-based ELISA assays is even poorer [26].

Based on all these considerations, the sizeable proportion of participants with positive *T. solium* antigen in both cohorts strongly suggests that active cysticercosis and neurocysticercosis are endemic in the study area, since false-positive results are infrequent for both conditions. Also, the high frequency of antigen positivity in patients with neurological disorders is compatible with the hypothesis that this infection plays some causal role [4, 32]. It is worth reminding in addition that neurocysticercosis tends to become clinically apparent when the cystic lesions degenerate, through local inflammatory reactions, while active cysts alone usually cause little neurological symptoms [35]. This may partly explain its age-dependent distribution.

Neuroimaging is absolutely necessary not only to accurately diagnose neurocysticercosis, but also to safely manage the potential risk of clinical deterioration due to anti-helminthic treatment (albendazole and/or praziquantel). While proven useful for epidemiological surveys and under study for assessing public health interventions (such as mass drug administration with anti-helminthic drugs in endemic regions), the utility of immunoassays is more questionable for clinical care, since no therapeutic decision can exclusively rely on their results [23, 35]. Antigen- or antibody-based assays, whenever available at the point-of-care [36, 37], could however be explored as an operational screening tool to select the subset of neurological patients who might benefit most from neuroimaging [38]. Since such investigations would be difficult to obtain and expensive in rural Congo, choosing assays or cutoffs with high specificity to diagnose active neurocysticercosis (the main form responsive to anti-helminthic treatment) needs to be prioritized, in order to minimize the number of unnecessary referrals. Prior to such a study however, adequate treatment of neurocysticercosis has to be made available in African low-resource hospitals [3].

## Conclusion

In this retrospective analysis of hospital-based neurology and persistent fever cohorts in the Kwilu province of DRC, *T. solium* antigen positivity was frequent (about 13 and 5%, respectively), confirming that this infection is prevalent in the region. Although the causal link with neurocysticercosis could not be formally established, our findings suggest that *T. solium* infection may contribute to the neurological case load in Central African areas. Further research should aim at performing brain imaging in neurological patients found with circulating antigens to fully characterize the spectrum of *T. solium*-associated morbidity in rural DRC, and at identifying risk factors for *T. solium* infection in order to design appropriate control measures targeting both the final and intermediate hosts.

## Data Availability

The Institute of Tropical Medicine of Antwerp, Belgium (sponsor of this study) is exploring the public repositories that allow making all material and data available with sufficient security. Meanwhile, the dataset is available on request.

## Abbreviations

CNS: Central Nervous System
CSF: Cerebrospinal fluid
DRC: Democratic republic of Congo, The
ELISA: Enzyme-Linked Immunosorbent assay
HGR: Hôpital Général de Référence
NIDIAG: Neglected Infectious diseases DIAGnosis
OD: Optical Density
